# Enzymatic Degradation of Multiple Major Mycotoxins by Dye-Decolorizing Peroxidase from *Bacillus subtilis*

**DOI:** 10.3390/toxins13060429

**Published:** 2021-06-19

**Authors:** Xing Qin, Xiaoyun Su, Tao Tu, Jie Zhang, Xiaolu Wang, Yaru Wang, Yuan Wang, Yingguo Bai, Bin Yao, Huiying Luo, Huoqing Huang

**Affiliations:** State Key Laboratory of Animal Nutrition, Institute of Animal Sciences, Chinese Academy of Agricultural Sciences, Beijing 100193, China; qinxing@caas.cn (X.Q.); suxiaoyun@caas.cn (X.S.); tutao@caas.cn (T.T.); zhangjie09@caas.cn (J.Z.); wangxiaolu@caas.cn (X.W.); wangyaru@caas.cn (Y.W.); wangyuan08@caas.cn (Y.W.); baiyingguo@caas.cn (Y.B.); yaobin@caas.cn (B.Y.)

**Keywords:** dye-decolorizing peroxidase, mycotoxin, aflatoxin B_1_, zearalenone, deoxynivalenol, biotransformation

## Abstract

The co-occurrence of multiple mycotoxins, including aflatoxin B_1_ (AFB_1_), zearalenone (ZEN) and deoxynivalenol (DON), widely exists in cereal-based animal feed and food. At present, most reported mycotoxins degrading enzymes target only a certain type of mycotoxins. Therefore, it is of great significance for mining enzymes involved in the simultaneous degradation of different types of mycotoxins. In this study, a dye-decolorizing peroxidase-encoding gene *BsDyP* from *Bacillus subtilis* SCK6 was cloned and expressed in *Escherichia coli* BL21/pG-Tf2. The purified recombinant *Bs*DyP was capable of oxidizing various substrates, including lignin phenolic model compounds 2,6-dimethylphenol and guaiacol, the substrate 2,2′-azino-bis (3-ethylbenzothiazoline-6-sulfonic acid), anthraquinone dye reactive blue 19 and azo dye reactive black 5, as well as Mn^2+^. In addition, *Bs*DyP could efficiently degrade different types of mycotoxins, including AFB_1_, ZEN and DON, in presence of Mn^2+^. More important, the toxicities of their corresponding enzymatic degradation products AFB_1_-diol, 15-OH-ZEN and C_15_H_18_O_8_ were significantly lower than AFB_1_, ZEN and DON. In summary, these results proved that *Bs*DyP was a promising candidate for the simultaneous degradation of multiple mycotoxins in animal feed and food.

## 1. Introduction

Mycotoxins are a diverse group of toxic secondary metabolites produced by certain filamentous fungi, and they display carcinogenicity, teratogenicity, immune toxicity, neurotoxicity, hepatotoxicity, nephrotoxicity, reproductive and developmental toxicity towards humans and animals [1]. Until now, more than 400 mycotoxins have been identified and reported; however, only a few mycotoxins dominate in contaminated food and animal feed. These are aflatoxins B_1_ (AFB_1_), zearalenone (ZEN), deoxynivalenol (DON), ochratoxin A, fumonisin B1, citrinin and patulin [2,3]. Meanwhile, the co-occurrence of abovementioned major mycotoxins is frequently observed in cereal-based animal feed and food [4,5], which might lead to synergistic and additive toxicological effects in humans and animals due to different mechanisms of action of mycotoxins at the cellular level [6].

The control of mycotoxins in food and feed depends on two main approaches: the prevention of mycotoxin production and detoxification of mycotoxins [7,8]. Most agronomic, physical and chemical strategies at pre-harvest as well as at post-harvest have been shown to be rather ineffective to control mycotoxin contamination [8]. With the increasing demand for effective approaches to reducing mycotoxin contamination, researchers have been paying more attention to biological strategies. The detoxification of mycotoxins through biotransformation by microorganisms or their enzymes has gradually become one of the most promising strategies because of its advantages in efficiency, specificity and environmental friendliness [7,9]. As for biotransformation of multiple mycotoxins, several microorganisms such as *Bacillus subtilis*, *Rhodococcus percolatus* and *Pseudomonas putida* have been reported to be capable of degrading AFB_1_ and ZEN [7,10,11,12]. In contrast, only a few enzymes have been identified, purified and characterized for multiple mycotoxin biotransformation.

Considering that part of mycotoxins is structurally similar to a lignin monomer or its derivatives, ligninolytic enzymes might be able to degrade different types of mycotoxins. In our previous study, we found that fungal manganese peroxidases could degrade four major mycotoxins, namely AFB_1_, ZEN, DON and fumonisin B_1_, in the presence of a dicarboxylic acid malonate [13]. Moreover, bacterial and fungal laccases could simultaneously degrade AFB_1_ and ZEN, using the laccase/mediator systems [14]. In this study, another ligninolytic enzyme dye-decolorizing peroxidase (DyP) from *B. subtilis* SCK6 was expressed in *Escherichia coli*, using the cold shock-inducible expression system coupled with co-expression of chaperones. Then the substrate specificity of purified recombinant *Bs*DyP was characterized. Moreover, the degrading capacity of three major mycotoxins AFB_1_, ZEN and DON by *Bs*DyP was evaluated, and corresponding enzymatic degradation products were identified by mass spectrometry analysis.

## 2. Results and Discussion

### 2.1. Gene Cloning, Expression and Purification of BsDyP from B. subtilis SCK6

It has been reported that *B. subtilis* could be able to efficiently degrade different types of mycotoxins, including AFB_1_ and ZEN [15,16]. However, very few mycotoxins-degrading enzymes are identified and characterized except for CotA laccase [14]. In the present study, genomic analysis of *B. subtilis* SCK6 revealed that ligninolytic enzymes were composed of DyP and laccase based on the PeroxiBase and CAZy databases annotation. One DyP gene, 1251 bp, was identified in the genome of *B. subtilis* SCK6, which encoded 416 amino acids with an estimated molecular weight of 45.7 kDa and an isoelectric point value of 8.71. The cloned *Bs*DyP from *B. subtilis* SCK6 shared 93% nucleic acid sequence identity and 96% amino acid identity with *Bs*DyP from *B. subtilis* KCTC2023 [17].

**Figure 1:**
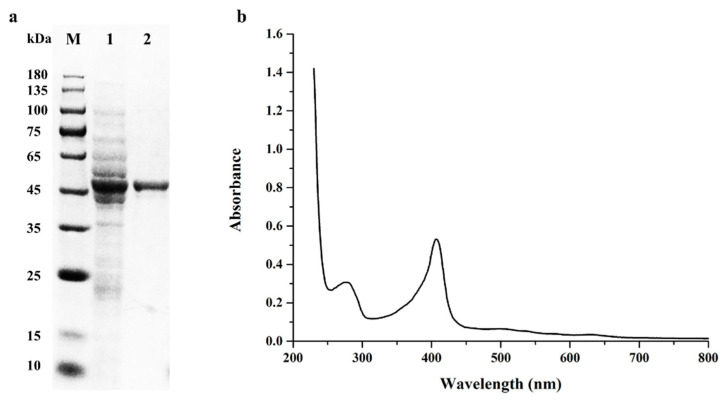
The analysis of purified recombinant *Bs*DyP by SDS-PAGE (**a**) and UV–visible spectroscopy (**b**). Lanes: M, the protein molecular mass marker; 1, the whole-cell lysis protein; 2, the purified recombinant *Bs*DyP.

*E. coli*, one of the most popular hosts for recombinant protein overexpression, was used as the expressing host to heterologously express *Bs*DyP. Given that the ligninolytic enzyme DyP expressed in *E. coli* accumulated in inclusion bodies which required another refolding process [18,19,20], the cold shock-inducible expression system coupled with co-expression of chaperones was applied to obtain the active DyP. With the help of molecular chaperones groES, groEL and tig, *Bs*DyP was successfully expressed in *E. coli* BL21 as a soluble form. A significant activity of DyP was detected in the sonicated cell supernatant, indicating the proper formation of disulfide bonds and heme incorporation into recombinant *Bs*DyP. After purification by immobilized metal affinity chromatography, *Bs*DyP showed a single band on SDS-PAGE gel, corresponding to the calculated molecular masses (Figure 1a).

### 2.2. Biochemical Characterization of the Purified Recombinant BsDyP

Like other ligninolytic peroxidases, including manganese peroxidase, versatile peroxidase and lignin peroxidase, DyP also showed the typical features of heme-containing enzymes with a Soret peak at 404–408 nm [21]. As shown in Figure 1b, *Bs*DyP had an obvious absorbance peak at 407 nm, indicating that the heme group was indeed incorporated into the purified recombinant *Bs*DyP. The Rz (A_407_/A_280_) ratio of the purified *Bs*DyP was 1.7.

**Figure 2:**
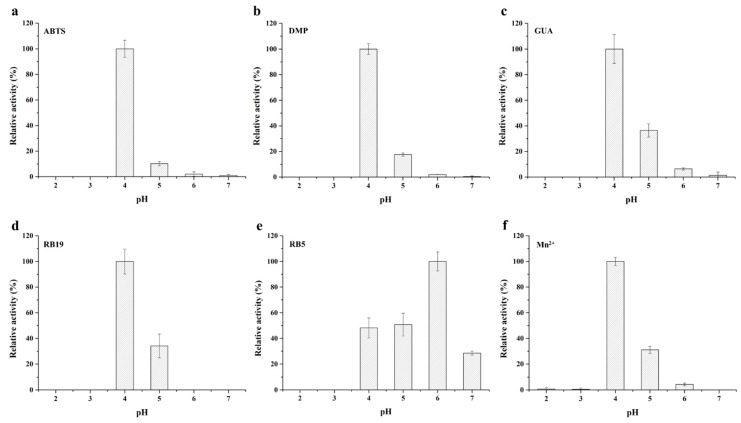
Optimum pH of the purified recombinant *Bs*DyP oxidizing different substrates: ABTS (**a**), DMP (**b**), GUA (**c**), RB19 (**d**), RB5 (**e**) and Mn^2+^ (**f**). Each assay was performed with three independent biological replicates.

It had been reported that DyP could act on a broad range of substrates, including lignin-derived phenolic and non-phenolic compounds, synthetic high redox potential anthraquinone and azo dyes [19,22]. In order to explore its substrate specificity, *Bs*DyP was tested on a series of lignin-derived compounds, synthetic dyes and Mn^2+^. The purified recombinant *Bs*DyP could oxidize the model substrate ABTS, phenolic compounds DMP and GUA, anthraquinone dye RB19, azo dye RB5 and Mn^2+^ except for the non-phenolic compound VA (Figure 2). Notably, *Bs*DyP had the ability to oxidize Mn2^+^ to Mn^3+^, which belongs to the catalytic properties of manganese peroxidase and versatile peroxidase [23,24]. The results suggested that *Bs*DyP might also contain the manganese binding sites.

In addition, the optimum pHs of the purified recombinant *Bs*DyP oxidizing various substrates, including ABTS, DMP, GUA, RB19 and Mn^2+^, were all 4.0, except that a higher optimum pH of *Bs*DyP against RB5 was 6.0. Meanwhile, *Bs*DyP was completely inactive when the pH was below 3.0, which was different from fungal DyP with a wider acidic pH range [22,25]. The specific activity of *Bs*DyP for ABTS, DMP, GUA, RB19 and Mn^2+^ at optimum pH were 7.25, 1.79, 0.03, 0.67 and 0.46 U/mg, respectively. Like other dye-decolorizing peroxidases, DyPs from *Pseudomonas putida*, *Thermobifida fusca* and *Thermomonospora curvata* showed higher specific activities towards ABTS and RB19, exhibiting two orders of magnitude higher than that of GUA [17,26,27]. However, the reason for difference in specific activities towards different substrates had not been well elucidated yet.

### 2.3. Degradation of Different Types of Mycotoxins by BsDyP

**Figure 3:**
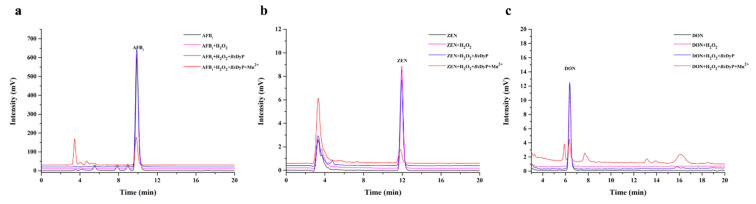
Degradation of different mycotoxins including AFB_1_ (**a**), ZEN (**b**) and DON (**c**) by 1.25 U/mL *Bs*DyP in 50 mM malonate buffer (pH 4.0) at 30 °C for 48 h.

Although DyP was reported to be involved in lignin degradation, industrial dyes decolorization [21,28], the great application value of DyP in xenobiotic bioremediation gradually attracted more and more attentions in recent years [29,30]. In this study, the different types of mycotoxins including AFB_1_, ZEN and DON were used to evaluate the mycotoxin degrading capability of *B*sDyP from *B. subtilis* SCK6. As shown in Figure 3, *Bs*DyP could efficiently degrade multiple mycotoxins in presence of Mn^2+^. AFB_1_, ZEN and DON were degraded up to 76.93%, 84.65% and 78.42% by the purified *Bs*DyP within 48 h, respectively. Unlike AFB_1_ and DON, ZEN also could be slightly degraded 11.78% by *Bs*DyP in absence of Mn^2+^, indicating that there might be two different mechanisms of ZEN degradation by *Bs*DyP. On one hand, it was similar to the detoxification mechanism of manganese peroxidases that oxidized Mn^3+^ and malonate acid mediated degradation of mycotoxins through the formation of radicals [13]. On the other hand, ZEN might be directly degraded by binding to the catalytic pocket of *Bs*DyP.

**Figure 4:**
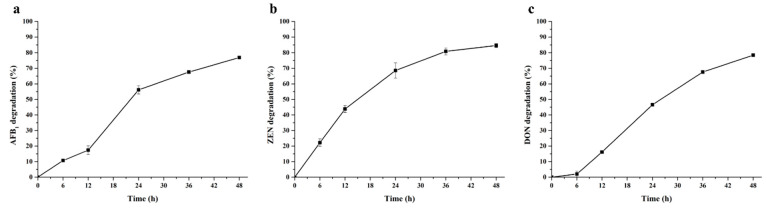
Time-course analysis of AFB1 (**a**), ZEN (**b**) and DON (**c**) by 1.25 U/mL BsDyP in 50 mM malonate buffer (pH 4.0) supplemented with 1 mM MnSO_4_ and 0.1 mM H_2_O_2_ at 30 °C. Each assay was performed with three independent biological replicates.

The time courses of AFB_1_, ZEN and DON degradation by *B*sDyP in presence of Mn^2+^ were shown in Figure 4. The degrading percentages of AFB_1_ and ZEN were 10.68% and 11.78% at 6 h, and then gradually ascended to 17.37% and 43.84% at 12 h. More than 50% AFB_1_ and ZEN degradation occurred after a 24 h incubation. In contrast, the initial degradation of DON was slower, with degradation percentages of 1.99% at 6 h, but DON content was significantly decreased at 12 h, and then 46.56% DON was degraded over 24 h of incubation.

### 2.4. Identification of AFB_1_, ZEN and DON Degradation Products

In order to further prove the effective detoxification of multiple mycotoxins by *Bs*DyP, it is necessary to identify the corresponding degradation products of mycotoxins and analyze their biological toxicities. Based on the clear relationship between chemical structure and biological activities of AFB_1_, ZEN and DON [31,32], the toxicology of degradation products could judge from chemical structure changes of different mycotoxins.

**Figure 5:**
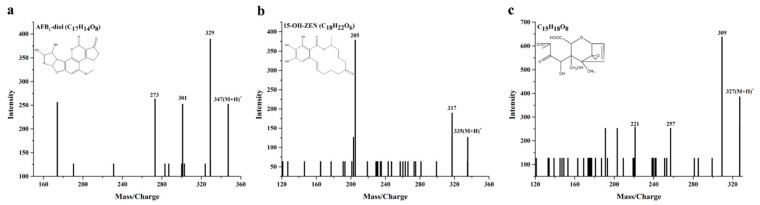
HPLC–MS/MS analysis of AFB1 (**a**), ZEN (**b**) and DON (**c**) degradation products by 1.25 U/mL BsDyP in 50 mM malonate buffer (pH 4.0) supplemented with 1 mM MnSO_4_ and 0.1 mM H_2_O_2_ at 30 °C for 48 h.

The main degradation product of AFB_1_ was identified by LC-MS/MS. The parent ion appeared at m/z 347.07 [M + H]^+^ and produced daughter ions of 329.06 [M − 18 + H]^+^, 301.07 [M − 46 + H]^+^ and 273.08 [M − 74 + H]^+^ (Figure 5a). These daughter ions were in accordance with the MS/MS fragments of AFB_1_-diol [33], suggesting that the C_8–9_ double bond of terminal furan rings in AFB_1_ was oxidized by *Bs*DyP and two hydroxyl groups were added to the molecular generate AFB_1_-diol. Remarkably, AFB_1_-diol was less toxic than AFB_1_ due to the removal of double bond in the terminal furan ring, which was considered as the basis for toxic and carcinogenic activity [32,34]. Similarly, the manganese peroxidase from the white rot fungus *Phanerochaete sordida* YK-624 could convert AFB_1_ to AFB_1_-diol and effectively remove the mutagenic activity of AFB_1_ [35]. Manganese peroxidases from the white rot fungus *Irpex lacteus* CD2 were able to oxidize AFB_1_ to AFB_1_-8,9-epoxide as the intermediate product [13]. Thus, the epoxidation and hydrolysis of AFB_1_ to produce AFB_1_-diol might be the common characteristic of AFB_1_ degradation by Mn^3+^ derived radicals.

The mass spectrum for the corresponding degradation product of ZEN is shown in Figure 5b. Daughter ions were obtained by fragmenting the parent ion with *m/z* 335.30 [M + H]^+^, including 317.15 [M − 18 + H]^+^ and 205.05 [M − 130 + H]^+^. These daughter ions were in agreement with the MS/MS fragments of 15-OH-ZEN [33], indicating that ZEN was hydroxylated at the C_15_ position of aromatic moiety by *Bs*DyP and 15-OH-ZEN was the major degradation product. The result revealed that there were significant differences between DyP and the well-known ZEN degrading enzyme lactonohydrolase ZHD101. Unlike the lactonohydrolase ZHD101, *Bs*DyP hydroxylated the aromatic ring rather than cleaved the ester bond of the macrolactone ring in ZEN [36]. Meanwhile, it had been reported that the hydroxylation of aromatic moiety in ZEN exhibited a significantly decreased estrogenicity and the estrogenicity of 15-OH-ZEN was remarkably decreased by 98% compared with ZEN [37]. These indicated that it might be a novel strategy feasible for biodetoxification of ZEN by *Bs*DyP from *B. subtilis* SCK6.

Although new product peaks of DON degradation by *Bs*DyP appeared in HPLC analysis, only one of the main degradation products was identified by LC–MS/MS, exhibiting a parent ion peak at m/z 327.11 [M + H]^+^. The corresponding daughter ions were *m/z* 309.09 [M − 18 + H]^+^, 257.10 [M − 70 + H]^+^ and 221.09 [M − 106 + H]^+^, which were consistent with the MS/MS fragments of C_15_H_18_O_8_ [31]. Although the enzymatic degradation mechanism of DON by *Bs*DyP was unclear, the possible structure of the degradation product C_15_H_18_O_8_ was shown in Figure 5c. According to structure-activity relationship studies, the toxicity of C_15_H_18_O_8_ might be significantly reduced due to the breakage of C_9–10_ double bond and the loss of C_3_-hydroxyl group [38]. To our knowledge, this is the first report about biotransformation of multiple mycotoxins, including AFB_1_, ZEN and DON by DyP. Moreover, the biological toxicities of each corresponding enzymatic degradation product, namely AFB1-diol, 15-OH-ZEN and C_15_H_18_O_8_, were significantly lower than AFB_1_, ZEN and DON. Though the other degradation products and their biological toxicity remain to be unveiled, these results might be of great value for practical application of *Bs*DyP in the biodetoxification of multiple mycotoxins in animal feed and food.

## 3. Conclusions

In this study, a dye-decolorizing peroxidase gene was cloned from *Bacillus subtilis* SCK6, and then successfully expressed in *E. coli* BL21 as a soluble form, using the cold shock-inducible expression system coupled with co-expression of chaperones. The recombinant *Bs*DyP could oxidize various lignin-derived phenolic compounds and high redox potential synthetic dyes, as well as Mn^2+^. Moreover, *Bs*DyP could efficiently degrade different types of mycotoxins, including AFB_1_, ZEN and DON, in the presence of Mn^2+^. The major degradation products of multiple mycotoxins were AFB_1_-diol, 15-OH-ZEN and C_15_H_18_O_8_, respectively. The predicted biological toxicity of each corresponding degradation product was significantly removed. Moreover, further studies are needed to confirm the detoxification effects for the pretreatment of contaminated food and animal feed. Nonetheless, the *Bs*DyP exhibited a great application potential for the enzymatic degradation of different types of mycotoxins in animal feed and food.

## 4. Material and Methods

### 4.1. Strain and Substrates

*B. subtilis* SCK6 was isolated and preserved in Institute of Animal Sciences, Chinese Academy of Agricultural Sciences, Beijing, China. Substrates 2,2′-azino-bis (3-ethylbenzothiazoline-6-sulfonic acid) (ABTS), 2,6-dimethylphenol (DMP), guaiacol (GUA), veratryl alcohol (VA), reactive blue 19 (RB19), reactive black 5 (RB5) and mycotoxins, including AFB_1_, ZEN and DON, were purchased from Sigma-Aldrich (St. Louis, MO, USA). Hemin was purchased from TCI (Tokyo, Japan).

### 4.2. Cloning and Expression of BsDyP

Based on the 5′ and 3′-end sequences of the *Bs*DyP structural gene, the *Bs*DyP was amplified with gene-specific primers (*Bs*DyP-NdeI-F: 5′ ATCATCATATCGAAGGTAGG*CATATG*AGCGATGAACAGAAAAAGC 3′; *Bs*DyP-XbaI-R: 5′ TTTTAAGCAGAGATTACCTA*TCTAGA*TGATTCCAGCAAACGCTG 3′). The PCR product was assembled into the linearized vector pCold I and then transformed into commercial *E. coli* BL21/pG-Tf2 chaperone competent cells (Takara, Japan), which harbored a plasmid pG-Tf2 encoding three molecular chaperones: groES, groEL and tig. The cells harboring pCold I-*Bs*DyP were in pre-cultured in LB medium supplemented with 100 μg/mL ampicillin and 20 μg/mL chloramphenicol at 37 °C overnight and used as the inocula of 300 mL LB medium containing 5 ng/mL tetracyclin, 100 μg/mL ampicillin and 20 μg/mL chloramphenicol. Tetracyclin was used as an inducer for the expression of molecular chaperones. The cultures were grown at 37 °C for 4 h, followed by the addition of 0.5 mM isopropy-β-d-thiogalactoside (IPTG) and 20 μM hemin for 12 h induction at 16 °C.

### 4.3. Purification and Characterization of BsDyP

After induction, the cells were harvested by centrifugation and resuspended in 30 mL binding buffer (20 mM sodium phosphate, 500 mM NaCl, pH 7.4). The cytosol containing the recombinant *Bs*DyP was released by sonication at 130 W for 30 min. *Bs*DyP was then purified using immobilized metal affinity chromatography with the washing buffer (20 mM sodium phosphate, 500 mM NaCl, 40 mM imidazole, pH 7.4) and elution buffer (20 mM sodium phosphate, 500 mM NaCl, 200 mM imidazole, pH 7.4). The purified recombinant *Bs*DyP was verified by SDS-PAGE, using 10% polyacrylamide gel. The protein content was determined by the Bradford method, using bovine serum albumin as the standard.

The DyP activity was measured by monitoring the oxidation of ABTS (ε_420_ = 36,000 M^−1^·cm^−1^) at 420 nm in the kinetic model by recording absorbance at 30 s intervals for 5 min, in a buffer containing 50 mM malonate, 1 mM ABTS and 0.1 mM H_2_O_2_ (pH 5.0 and 25 °C). One unit of enzyme activity was defined as the amount of enzyme that oxidizes 1 μmol of ABTS per min at 25 °C. The purified recombinant *Bs*DyP was first subjected to UV–visible spectroscopic analysis in the range of 230 to 800 nm in the 20 mM pH 5.0 malonate buffer. Then the substrate specificity of *Bs*DyP was studied for the oxidation of different substrates, including ABTS, DMP (ε_470_ = 12,100 M^−1^·cm^−1^), GUA (ε_465_ = 49,600 M^−1^·cm^−1^), VA (ε_310_ = 9300 M^−1^·cm^−1^), RB19 (ε_595_ = 10,000 M^−1^·cm^−1^), RB5 and Mn^2+^ (ε_270_ = 11,590 M^−1^·cm^−1^), in 50 mM sodium malonate buffers with pH values ranging from 2.0 to 7.0 at 25 °C. The maximum activity of *Bs*DyP was considered to be 100%.

### 4.4. Degradation of Different Types of Mycotoxins

*Bs*DyP (1.25 U/mL) was incubated with different types of mycotoxins (AFB_1_ and ZEN 1 µg/mL; DON 10 µg/mL) in 50 mM pH 4.0 malonate buffer supplemented with 0.1 mM H_2_O_2_ in the presence or absence of 1 mM MnSO_4_. The reaction was carried out at 30 °C for 48 h. Periodically, samples were taken out and equal volume methanol were added to stop the reaction.

HPLC analysis of AFB_1_, ZEN and DON degradation were performed by using a SHIMADZU 20A series instrument (Kyoto, Japan) equipped with a UV/Vis detector and RF-20A fluorescence detector with a Waters XBridge C18 column (5 µm, 4.6 mm × 150 mm). The elution condition for AFB_1_ was set as follows: 45% methanol at a flow rate of 0.8 mL/min; AFB_1_ was monitored at 360 nm excitation wavelength and 440 nm emission wavelength. The elution condition for ZEN was set as follows: 45% acetonitrile (ACN) at a flow rate of 0.8 mL/min; ZEN was monitored at 274 nm excitation wavelength and 440 nm emission wavelength. The elution condition for DON was set as follows: 10% ACN at a flow rate of 0.8 mL/min; DON were monitored at 218 nm.

AFB_1_, ZEN and DON degradation products were further analyzed by using LC–MS/MS, which was carried out by coupling a SHIMADZU Nexera UHPLC system (Kyoto, Japan) to an AB-SCIEX 5600+ Triple TOF mass spectrometer. For LC analysis, the elution program was as follows: 0–70% ACN, 7 min; 70–100% ACN, 5 min; and 100% ACN, 1 min. For MS analysis, the parameters were set as ion source gases GS1 and GS2 and curtain gas, at 55, 55 and 25 psi, respectively; temperature was 500 °C; and ion spray voltage floating was at 5500 V.

## Data Availability

The data presented in this study are available on request from the corresponding author.
